# Cellular therapeutics and immunotherapies in wound healing – on the pulse of time?

**DOI:** 10.1186/s40779-024-00528-5

**Published:** 2024-04-18

**Authors:** Lioba Huelsboemer, Leonard Knoedler, Alejandro Kochen, Catherine T. Yu, Helia Hosseini, Katharina S. Hollmann, Ashley E. Choi, Viola A. Stögner, Samuel Knoedler, Henry C. Hsia, Bohdan Pomahac, Martin Kauke-Navarro

**Affiliations:** 1 Division of Reconstructive and Plastic Surgery, Yale School of Medicine, New Haven, CT 06510 USA; 2 School of Medicine, University of Regensburg, 93040 Regensburg, Germany; 3 Regenerative Wound Healing Center, Yale School of Medicine, New Haven, CT 06510 USA; 4 School of Medicine, University of Wuerzburg, 97070 Würzburg, Germany; 5 Department of Pathology, Massachusetts General Hospital, Harvard Medical School, Boston, MA 02114 USA; 6 California University of Science and Medicine, Colton, CA 92324 USA

**Keywords:** Chronic wounds, Wound healing, Wound healing disorder, Biofilm, Immunotherapies, Cellular therapies

## Abstract

Chronic, non-healing wounds represent a significant challenge for healthcare systems worldwide, often requiring significant human and financial resources. Chronic wounds arise from the complex interplay of underlying comorbidities, such as diabetes or vascular diseases, lifestyle factors, and genetic risk profiles which may predispose extremities to local ischemia. Injuries are further exacerbated by bacterial colonization and the formation of biofilms. Infection, consequently, perpetuates a chronic inflammatory microenvironment, preventing the progression and completion of normal wound healing. The current standard of care (SOC) for chronic wounds involves surgical debridement along with localized wound irrigation, which requires inpatient care under general anesthesia. This could be followed by, if necessary, defect coverage via a reconstructive ladder utilizing wound debridement along with skin graft, local, or free flap techniques once the wound conditions are stabilized and adequate blood supply is restored. To promote physiological wound healing, a variety of approaches have been subjected to translational research. Beyond conventional wound healing drugs and devices that currently supplement treatments, cellular and immunotherapies have emerged as promising therapeutics that can behave as tailored therapies with cell- or molecule-specific wound healing properties. However, in contrast to the clinical omnipresence of chronic wound healing disorders, there remains a shortage of studies condensing the current body of evidence on cellular therapies and immunotherapies for chronic wounds. This review provides a comprehensive exploration of current therapies, experimental approaches, and translational studies, offering insights into their efficacy and limitations. Ultimately, we hope this line of research may serve as an evidence-based foundation to guide further experimental and translational approaches and optimize patient care long-term.

## Background

**Fig. 1:**
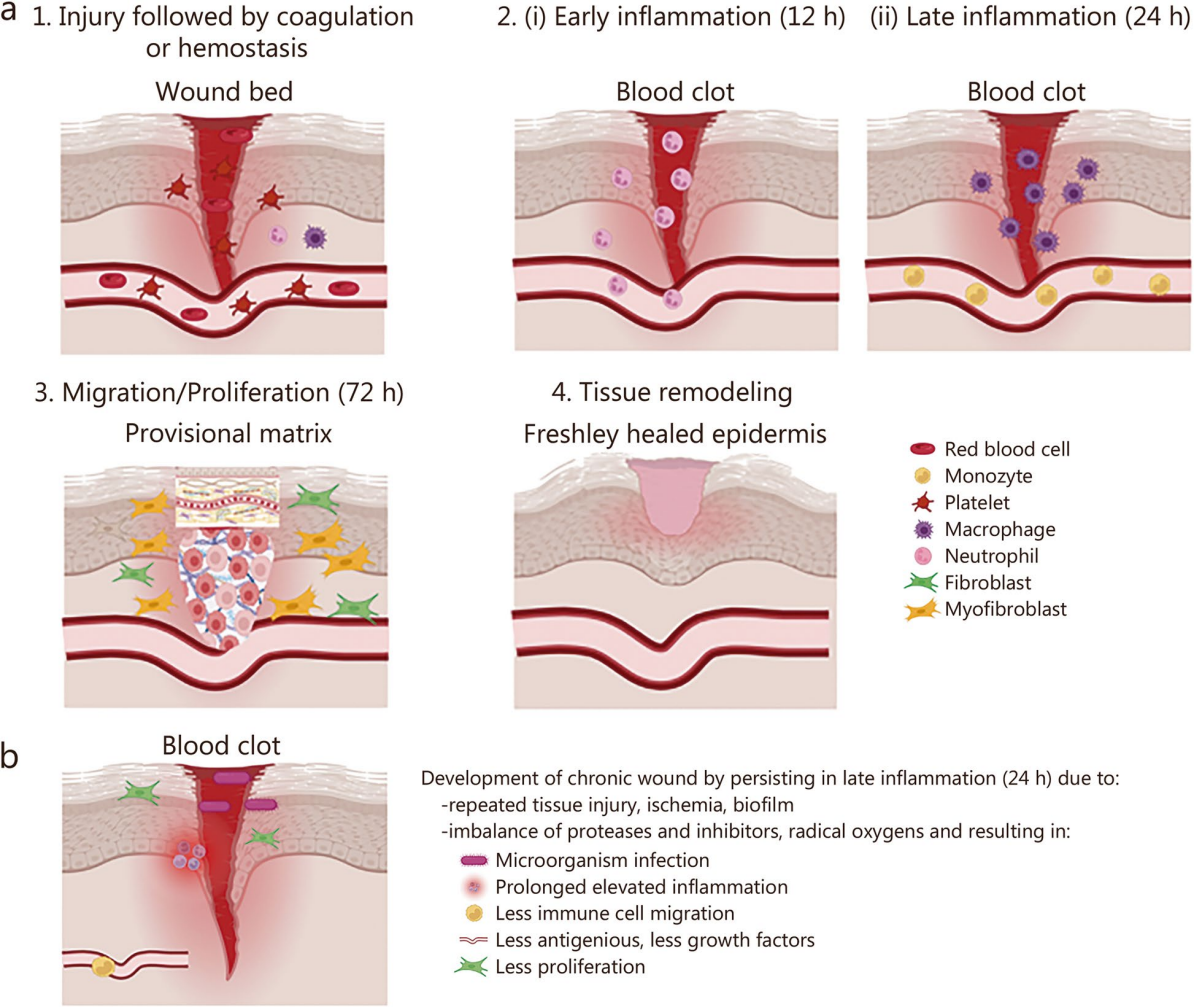
Schematic diagram of physiological wound healing stages (**a**) and development of chronic wound due to persistence in inflammatory stage of wound healing (**b**). Created with Biorender.com

Demographic changes pose significant challenges for healthcare systems across the globe. Advancing age is often accompanied by the prevalence of underlying health conditions, such as diabetes and arteriosclerosis, which contribute to the higher incidence of chronic wound healing disorders [1]. For example, a retrospective analysis of Medicare beneficiaries in the United States revealed that a substantial number of individuals, up to 8.2 million patients (14.5%), were affected by chronic wound disorders [2]. Of particular concern is the finding that 5-year mortality rates of patients suffering from diabetic foot complications are comparable to the overall rates observed in cancer-associated cases (31%) [3]. Ordinarily, a wound heals damaged tissue through 4 sequential, overlapping phases: coagulation or hemostasis, early and late inflammation, proliferation, and tissue remodeling (Fig. 1a). These steps occur in a timely and highly orchestrated manner that involves the activity and recruitment of myriad cell types and ultimately culminates in the restoration of tissue homeostasis via the regeneration of functional tissue or, as is most often the case, scar tissue. Whereas normal wounds are able to resolve, chronic wounds are defined as wounds that have failed to progress toward healing after 4 weeks of standard of care (SOC) treatment [4]. This occurs because chronic wounds are unable to properly progress through the 4 aforementioned phases, precluding the restoration of a functional barrier and making them amenable to infection and tissue death. Despite etiologies ranging from vascular insufficiency to pressure or diabetic complications, the underlying physiology is typically unifying across all types: arrest in the inflammatory phase of wound healing, characterized by excessive inflammatory signaling, increased protease activity, abundant reactive oxygen species, and a deficiency of growth factors and stem cell activity [5]. Other factors may also contribute to deficient wound healing and chronic wound formation, including attenuated proliferation, inadequate angiogenesis, and microorganism colonization (Fig. 1b). Non-healing wounds have also been found to have significantly lower oxygen tension levels, ranging from 5 to 20 mmHg, whereas proper wound healing requires a tissue oxygen tension above 25 mmHg [6, 7]. Conditions like diabetes or vascular disorders can further contribute to a reduced peripheral oxygen supply. A considerable proportion (78%) of chronic wounds is affected by the presence of biofilms, structured communities of microorganisms embedded within an extracellular polymeric substance that often forms in wound beds. This state allows microorganisms to become highly tolerant and resistant to the host’s immune system and antimicrobial substances [8, 9], posing a significant challenge to treatment and recovery [10]. To effectively promote wound healing, then, evidence-based strategies must be adequately identified. However, there remains a paucity of studies that comprehensively summarize the existing body of evidence on this subject. The current SOC management across all types of chronic wounds includes initial surgical debridement of damaged or necrotic tissue followed by the decision to either allow the wound to heal by secondary intention or grafting tissue onto the wound bed. Either choice is then supported by the use of local or systemic antibiotic treatment and the application of an appropriate wound dressing. It should be noted that different approaches and techniques exist within each of these steps [4].

Exploring this line of research has the potential to customize therapeutic strategies and achieve more targeted effects while minimizing systemic side effects. Below, we explore the current literature regarding available or developing cellular therapies that employ regulatory T-cells (T-regs), stem cells, macrophages, fibroblasts, and platelets in addition to immunotherapies for chronic, non-healing wounds (Table 1). Moreover, understanding the mechanisms of wound healing and their interactions with underlying diseases is crucial for the development of effective therapeutic interventions.

**Table 1:** Comprehensive outline of mechanisms, available human trials data as well as limitations of the here proposed studies

Approach	Therapeutic	Proposed mechanisms/results	Application	Human trials data	Limitations	References
Regulatory T cells (T-regs)	Not applicable (N/A)	Pre-clinical evidence suggests that T-regs may act as a therapeutic for non-healing wounds by helping terminate the inflammatory phase. This would allow for the wound healing sequence to progress and tissue recovery and homeostasis to be achieved	Pre-clinical murine model	N/A	The functional application of T-reg-based therapies has only been explored in vitro and pre-clinical models. Extensive research is still required to determine their use in humans	[11–13]
Induced pluripotent stem cells (iPSCs)	N/A	The clinical application of iPSCs lies in their ability to transform into fibroblasts, keratinocytes, endothelial cells, dermal stem cells, and a variety of different cell types. iPSCs may be used to culture and then transplant depleted cell types within chronic wounds, or potentially be directly applied to wound beds and behave as a source of growth factors, depleted cell types, and as orchestrators of inflammation-resolving programs	Pre-clinical murine model	N/A	The major concern with the clinical application of iPSCs is their safety profile, especially regarding their tumorigenic/teratogenic potential	[14, 15]
iPSC-derived smooth muscle cells	N/A	One specific study evaluated human iPSC-derived smooth muscle cells. The group found that application accelerates diabetic wound healing to a greater extent than acellular and adipose-derived stem cell-containing scaffolds	Pre-clinical murine model	N/A	In the specific iPSC-derived smooth muscle cell study, the authors mention the streptozotocin model of diabetes approximating type 1 diabetes more than type 2 diabetes, which is the patient demographic that mostly suffers from chronic, non-healing wounds. Moreover, only male mice were used for the study	[14, 15]
Stem cell-based therapies	Grafix	Grafix is a cryopreserved amniotic membrane with a 3D-extracellular matrix (ECM) scaffold that contains growth factors, fibroblasts, endothelial cells, and mesenchymal stem cells (MSCs). The use of MSCs is unique to Grafix and although the authors acknowledge that the mechanism remains to be elucidated, they cite data that suggests MSCs may play an important role in all phases of wound healing and complete tissue recovery	Human trials	Food and Drug Administration (FDA)-approved with indications for chronic wounds	Clinical trials of Grafix have not been updated since 2016. Moreover, although one of the studies was a robust randomized controlled trial (RCT), the other was a retrospective study that lacked a proper control group. Finally, there may have been variability in data reporting amongst the participating clinics	[16]
Pharmaceutical macrophage therapy	Infliximab	Infliximab is a monoclonal, neutralizing antibody of tumor necrosis factor-α (TNF-α). Studies have shown that inhibition of TNF-α prevents M1 macrophage polarization and activity. This suppresses the inflammatory microenvironment and allows for normal wound healing progression to continue	Human trials	Infliximab is FDA-approved with indications for a variety of autoimmune diseases, including Crohn’s disease, ulcerative colitis, rheumatoid arthritis, psoriatic arthritis, and plaque psoriasis, among others. However, it is not FDA-approved nor indicated for the treatment of chronic wounds	The study referenced is from 2006. Moreover, the study design was not completely robust, as it was unblinded and contained a small sample size	[17]
	GQ-11	GQ-11 is a partial/dual peroxisome proliferator-activated receptor α/γ (PPARα/γ) agonist. Activation of PPARα is postulated to promote anti-inflammatory phenotypes in activated M1 macrophages by repressing pro-inflammatory gene expression and inducing anti-inflammatory gene expression	Pre-clinical murine model	N/A	Although there was an overall benefit with GQ-11 treatment, effect sizes were statistically significant but modest. Moreover, treatment with GQ-11 did not show any effect in non-diabetic mice	[18]
Growth factor therapy	Beclapermin	Beclapermin is a topical formulation of recombinant human platelet-derived growth factor subunit B (PDGF-BB). It promotes the recruitment and proliferation of fibroblasts and immune cells to the wound bed, promoting granulation tissue formation	Human trials	FDA-approved with indications for diabetic, chronic wounds	The original clinical study is from 1999. No updated study was found. Moreover, its use has not been established in pressure or venous stasis ulcers	[19]
Combined cellular therapies	Dermagraft	Dermagraft is a sterile, cryopreserved, human fibroblast-derived dermal substitute. It is generated from cultured neonatal dermal fibroblasts onto a mesh scaffold. It does not contain any other cell type. It is thought to work by serving as a source of cytokines, growth factors, and living cells, which can then migrate into the wound bed and promote granulation tissue formation	Human trials	FDA-approved with indications for chronic wounds	The RCT was designed using Dermagraft in conjunction with, and not as a replacement for, the standard of care (SOC) of debridement, disinfection, and bandaging	[20]
	Apligraf	Apligraf is a bi-layered bioengineered skin substitute constructed from neonatal fibroblasts and keratinocytes derived from human foreskin. Although its exact mechanism is unknown, it is thought to provide cytokines and growth factors to aid in wound healing	Human trials	FDA-approved with indications for chronic wounds	The RCT was designed using Apligraf in conjunction with, and not as a replacement for, the SOC of debridement, disinfection, and bandaging. Moreover, there may have been variability in data reporting amongst the participating clinics. However, 17/33 (51.5%) of Apligraf-treated patients did report full wound closure compared to 10/38 (26.3%) of control patients	[21]
	TheraSkin	TheraSkin is an all-human, split-thickness, skin allograft that is procured from human skin donors. It is thought to work by serving as a source of endogenous growth factors and cells in addition to providing a native ECM. Moreover, TheraSkin can be vascularized by the recipient following transplantation to support the development of granulation tissue, which aids in epithelialization to support wound closure	Human trials	FDA-approved with indications for chronic wounds	The study was designed using TheraSkin in conjunction with, and not as a replacement for, the SOC of debridement, disinfection, and bandaging. There also may have been variability in data reporting amongst the participating clinics. Finally, it was a retrospective-matched cohort study which could have overlooked potential confounders	[22]
Platelet-rich fibrin (PRF)	Leukocyte-rich fibrin and PRF	PRF is a biodegradable fibrin scaffold embedded with large numbers of leukocytes and platelets. The entrapment of cells within a scaffold is thought to be superior to platelet-rich plasma due to prolonged and regulated release of growth factors that aid in wound healing	Human trials	Does not require FDA-approved as it is derived from a patient’s blood with only physical modifications. Use on chronic wounds is dependent on the provider’s discretion	The study referenced was self-controlled with limited strength. However, a systemic review shows that 18 of 31 clinical studies reported positive wound healing outcomes compared to control	[23, 24]
Platelet extracellular vesicles (PEVs)	N/A	PEVs are released from platelets once activated. They contain stores of growth factors, cytokines, and ECM modulators. Studies have demonstrated how PEVs are able to aid in wound recovery by stimulating proliferation and angiogenesis	Human trials	N/A	Unfortunately, this study only focused on safety and not specific outcomes. The PRF control had better wound healing outcomes than the PEV treatment group. However, the authors noted that further research into the most effective dose is warranted	[25]
Polyclonal antibodies	N/A	Polyclonal antibodies are used, for example, to target the N-terminal of *Pseudomonas aeruginosa* (*P. aeruginosa*) type b flagellin in infected burn wounds and also to inactivate pathogenic toxins	Pre-clinical murine model	N/A	The study was only pre-clinical and had a small sample size. It should be noted that there was no difference in mortality and wound healing time between anti-*P. aeruginosa* antibody treatment and imipenem antibiotic treatment	[26]
Monoclonal antibodies	N/A	Topical programmed death-ligand 1 (PD-L1) is a checkpoint inhibitor that can, applied as a gel, support re-epithelialization and attenuating prolonged inflammation	Pre-clinical murine model	N/A	The study was only pre-clinical and had a small sample size	[27]
Wnt inhibitor	Pyrvinium	Pyrvinium, at least in pre-clinical studies, may enhance MSC proliferation, engraftment, and stemness in an in-vitro and in-vivo wound healing model. Pyrvinium was separately found to increase vascularity, cellular proliferation, and general organization of granulation tissue in a polyvinyl alcohol (PVA) sponge model. These results suggest it may be used as an ancillary therapy to stem cell-based therapies	Pre-clinical murine model	Pyrvinium pamoate is FDA-approved with indications as an anthelmintic. A single ongoing phase I trial is being conducted to evaluate its utility in pancreatic cancer (PMID: 34,413,127). It is not currently indicated for the treatment of chronic wounds	Only in vitro and pre-clinical data is available which demonstrates statistically significant but relatively small effect sizes. Moreover, our search found only one group that has researched its potential therapeutic use in wound healing	[28, 29]
Hypoxia inducible factor-1α (HIF-1α)	Deferoxamine (DFO)	DFO is an iron chelator. HIF-1-prolyl hydroxylase (PHD), an enzyme responsible for HIF-1 degradation, requires iron as a cofactor. Chelation of iron then indirectly inhibits HIF-1-PHD, increasing the abundance of HIF-1 which leads to increased vascular endothelial growth factor (VEGF) expression	Pre-clinical murine model	DFO is FDA-approved with indications for iron overload and is not currently indicated for the treatment of chronic wounds	The study was only pre-clinical and had a small sample size. Moreover, effect sizes were statistically significant but relatively small	[30, 31]
	Cyclometalated iridium (III) metal complex 1a	Cyclometalated iridium (III) metal complex 1a functions as a stabilizer of HIF-1α. It does so by disrupting the von Hippel-Lindau-HIF-1α protein interaction, allowing for the accumulation of HIF-1 in cellulose. Wild-type and diabetic mice with excisional wounds treated with cyclometalated iridium (III) metal complex 1a showed an accelerated rate of wound healing accompanied by increased skin thickness, collagen deposition, and, evidence of neovascularization	Pre-clinical murine model	N/A	We only found the recent study by Li et al. [32] that used cyclometalated iridium (III) metal complex 1a to accelerate diabetic wound healing. Moreover, the authors report that their murine models all have limitations and cannot fully replicate the human diabetic pathological state	[32]
Antibiofilm therapies	Antibody-mitomycin C conjugate	Antibody-antibiotic conjugates have become important objects of research due to their specificity and increased bacterial killing compared to standalone antibiotic treatment. Mitomycin C is an antibiotic used as an anti-neoplastic agent. In vitro, however, mitomycin C is also a potent anti-bacterial and biofilm eradicator	Pre-clinical murine model	Mitomycin C is FDA-approved with indications for gastric and pancreatic adenocarcinoma as well as urothelial cancer. It is not currently indicated for use as an anti-biofilm antibacterial	The study did not find a significant difference in bactericidal effect between antibody-conjugated mitomycin C and mitomycin C alone. However, two counter points were proposed by the authors. First, toxicity was lower in conjugate treatment. Second, the maximum effective dose of the antibody-antibiotic conjugate was not explored, suggesting a higher dose may be more effective	[33]
	Rifampicin-containing nanoparticles	Polymeric nanoparticles loaded with rifampicin and covalently bound to a *Staphylococcus aureus* (*S. aureus*)-specific antibody was demonstrated to accumulate in *S. aureus* biofilms and have significantly increased bactericidal effects against both planktonic and biofilm bacteria compared to free-form rifampicin	Pre-clinical murine model	N/A	The study only used 3 mice per treatment group (*n* = 12 total), although the effect sizes of the antibody-conjugate treatments were considerably large	[34]

## Cellular therapies for wound healing

### T-regs

T-regs are a heterogeneous subset of the cluster of differentiation (CD)4^+^ helper T-cells that maintain immune tissue homeostasis by promoting self-tolerance, dampening excessive immune responses, and suppressing autoimmunity [35–37]. They are classically defined by the expression of CD4, CD25, and forkhead box protein 3 [35, 36, 38]. Due to their heterogeneity, T-regs exert their immune tolerance and suppression via multiple mechanisms: secretion of anti-inflammatory factors such as interleukin (IL)-10, transforming growth factor-β (TGF-β), and IL-35, suppression of tumor necrosis factor-α (TNF-α), IL-6, and interferon (IFN)-γ release, and the quelling of T-cell activity and proliferation via cytotoxic T-lymphocyte antigen-4 [36, 39].

Within the context of wound healing, it is theorized that T-regs may aid non-healing wounds in terminating the inflammatory phase and enabling progression to the proliferative phase to continue the wound healing sequence [11]. Lewkowicz et al*.* [40] indicated that lipopolysaccharide-activated T-regs can directly inhibit the inflammatory activity of polymorphonuclear neutrophils by inducing their production of IL-10, TGF-β, and heme oxygenase-1 generation while inhibiting the production of IL-6 in vitro. Moreover, T-regs have been shown to directly suppress the inflammatory activity of macrophages in vitro by hampering their ability to secrete TNF-α and IL-6, in addition to reducing their recruitment [41]. The anti-inflammatory activity is likely stimulated by epidermal growth factor (EGF), as Zaiss et al*.* [42] indicated how T-reg suppressive activity is largely enhanced when they are exposed to EGF.

These positive findings have also been observed in pre-clinical, animal models. Nosbaum et al*.* [11] used an in vivo murine model to highlight the necessity of T-regs for appropriate wound closure. The group found that T-regs were activated and preferentially accumulate in wounded skin during the inflammatory phase of wound healing while depletion of T-regs significantly diminished wound closure. T-regs mediated this effect by attenuating IFN-γ-secreting effector T-cells and, consequently, the accumulation of pro-inflammatory LyC6^+^ macrophages in the wound. Similarly, Haertel et al. [13] found that depletion of T-regs caused poor vessel formation, reduced contraction, and obstructed reepithelization in both wild-type and activin-transgenic mice. These findings strongly support the necessary role of T-regs in normal wound healing. Furthermore, these data suggest that the topical administration of T-reg-based cell therapy could be promising for superficial and deep tissue chronic wounds, particularly for those wounds that persist in the inflammatory phase due to T-regs’ role in anti-inflammatory processes.

However, although pre-clinical studies are enlightening, significantly more basic science research regarding the precise role of T-regs in wound healing and the mechanism by which they mediate it is required. Knoedler et al. [43] discussed future directions of T-regs, noting that chimeric antigen receptor-equipped T-regs may deliver promising treatment options for wound healing due to chimeric antigen receptor-T-regs proven track record in controlling alloimmune-mediated rejection in human skin grafts. However, potential side-effects such as T-reg overstimulation or exhaustion have to be further investigated pre-clinical prior to clinical application.

With all this in mind, the limitations of adoptive T-reg cell therapies include the use of autologous cells from patients with chronic wounds whose T-regs may be dysfunctional, the time-consuming expansion of T-regs in vitro*,* cell delivery and survival in the inflammatory wound environment, and lack of standard generation protocols [44]. Consequently, there are currently no registered clinical trials nor Food and Drug Administration (FDA)-approved therapies for the use of adoptive T-reg cell therapy for the treatment of chronic, non-healing wounds.

### Stem cells

A stem cell is defined as an immature, unspecialized cell that can undergo self-propagation and has the ability to differentiate into more than one cell lineage, often to replace aged or damaged tissue [45, 46]. Two fundamental ideas position stem cells as a promising therapeutic prospect for the treatment of chronic wounds. First, the ability of stem cells to spatially and temporally respond to fluctuating microenvironments by secreting growth factors and differentiating into depleted cell types suggests that they may be more effective at restoring homeostasis than other therapies. This is particularly important as the presence of hypoxia, poor perfusion, microbial growth, and inflammation generally precludes many therapies from being effective [16, 47, 48]. Second, the observation that stem cell populations are depleted in non-healing wounds supports the notion that their replacement may be advantageous [47, 48].

Pluripotent stem cells (PSCs) are generally divided into two categories: embryonic stem cells (ESCs) and induced PSCs (iPSCs). ESCs are PSCs derived from the inner cell mass of a blastocyst and have the capacity to differentiate into any tissue of the three germ layers [49, 50]. However, due to the controversial ethical dilemma surrounding ESC research and clinical applicability, iPSCs have become their functional replacement.

**Fig. 2:**
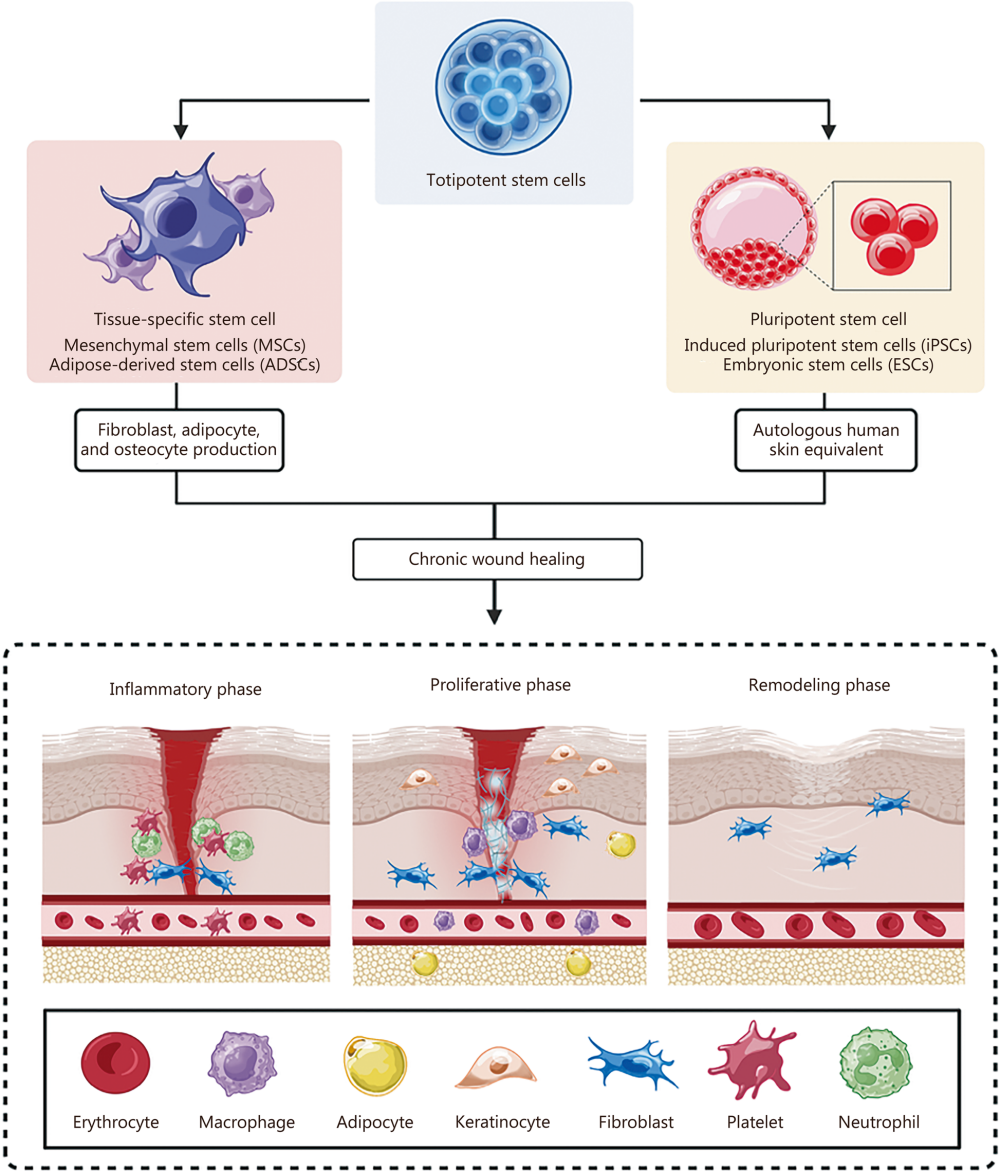
Totipotent stem cells can differentiate into tissue-specific and pluripotent stem cells that promote chronic wound healing via different pathways. Created with Biorender.com

iPSCs, which phenotypically resemble ESCs, are PSCs capable of differentiating into any tissue of the three germ layers. Unlike ESCs, however, they are derived by reprogramming adult somatic cells (most commonly keratinocytes or fibroblasts) with a cocktail of four transcription factors [51]. Because iPSCs are easy to generate and present low immunogenicity when self-sourced, they possess all the benefits of ESCs while avoiding the moral dilemma. Christiano et al. [52] used iPSCs to generate an autologous 3D human skin equivalent for wound healing, surgical reconstruction, or skin diseases. And Takagi et al. [53] developed an artificial, 3D allograft from iPSCs which contained functional skin appendages such as hair follicles and sebaceous glands. In fact, cells derived from iPSCs may contribute to extracellular matrix (ECM) deposition, angiogenesis, cell proliferation, and immunoregulation [14]. Sasson et al. [54] have investigated TNF-α preconditioned human iPSC in terms of cellular viability and secretome when integrated into a 3D collagen scaffold. Their results showed improved cell viability as well as a significant increase in vascular endothelial growth factor (VEGF) expression in the preconditioned human iPSCs meaning that augmented cellular viability in combination with secretion of paracrine factors could lead to improved wound healing due to migration (Fig. 2).

Despite the promising landscape, there are currently no ESC or iPSC therapies that are commercially available for non-healing wounds or, in fact, for any disease. As mentioned previously, ESCs are unlikely to see clinical application due to ethical considerations. And while significant progress has been made in characterizing iPSCs and their application, their safety profile remains uncertain for clinical use, particularly due to their tumorigenic potential [15]. Furthermore, determining the optimal platform for delivering iPSCs to chronic wounds and characterizing the ideal microenvironment to ensure their survival is crucial. Innovative approaches to eliminate undifferentiated cells that possess tumorigenic potential before cellular transplantation are also required. Finally, there are considerations to be made with iPSCs derived from patients with different health profiles. We conclude that primate studies are likely necessary before iPSCs can make the jump towards clinical use in humans [15].

Adult stem cells (ASCs) also known as tissue-specific stem cells, are stem cells that reside in and can be isolated from non-fetal tissue [55]. In contrast to PSCs, ASCs are multipotent, meaning their progeny is restricted to the lineage of a single layer [56]. One of the principal roles of ASCs, then, is to maintain the integrity of the tissues they reside in by replacing old or damaged cells.

Although many distinct types of ASCs exist, mesenchymal stem cells (MSCs) dominate the stem cell-based therapy field with regard to non-healing wound treatment. MSCs are of mesodermal origin and are found in many tissues throughout the body, including bone marrow, adipose tissue, umbilical cord blood, Wharton’s Jelly, and many other tissues. Bian et al. [57] present a comprehensive review of the now-established effects that MSCs exert in vitro, including the secretion of proliferative and angiogenic growth factors, exerting anti-inflammatory and immunoregulatory effects, and differentiating into mesodermal lineage cell types such as fibroblasts, keratinocytes, and endothelial cells.

The two most prevalent types of MSCs in wound healing studies are bone marrow mesenchymal stem cells (BMSCs) and adipose-derived stem cells (ADSCs) (Fig. 2). Falanga et al. [58] had previously reported that topically-applied autologous BMSCs were able to significantly accelerate and improve wound healing in both non-healing human wounds and diabetic murine models. However, the difficulty in sourcing BMSCs, combined with reduced red marrow content as patients age, has made finding an alternative source of ASCs compelling [59]. ADSCs have consequently become an increasingly relevant object of research as they are abundant in adipose tissue, easily harvested from lipoaspirates, and not subject to the same limitations as BMSCs [59]. Brembilla et al. [60] provide an excellent review of both pre-clinical and clinical data which supports the idea that ADSCs can improve reepithelization, promote angiogenesis, and accelerate wound healing. However, they also highlight the need for further research and well-designed clinical trials before their effectiveness can be conclusively appraised.

To date, the only FDA-approved, stem cell-based therapy for chronic wounds is Grafix, a cryopreserved amniotic membrane with a 3D-ECM scaffold containing growth factors, fibroblasts, endothelial cells, and MSCs [16, 49]. Two clinical studies, including a large randomized controlled trial (RCT), demonstrate the safety and efficacy of Grafix treatment (*n* = 50 and *n* = 66) in diabetic foot ulcers, even in those that are treatment refractory. Patients who were treated with Grafix achieved wound closure (62%) up to 4 weeks faster than the SOC-treated patients (21.3%) while also experiencing fewer complications [16, 49].

Ultimately, stem cell therapies are a promising avenue for chronic wound treatment, with the current literature largely focusing on MSC-based treatments [61]. However, although several registered stem cell clinical trials are underway, the dearth of commercial drugs clearly suggests further research is necessary [26].

### Macrophages

Macrophages are long-lived phagocytic cells of the innate immune system that play specialized roles in immune protection, inflammation, and tissue homeostasis [62]. Tissue-resident macrophages of the skin are broadly divided into two categories: epidermal macrophages (also known as Langerhans cells) and dermal macrophages [63]. Monocyte-derived macrophages are also recruited during periods of infection, inflammation, or injury, serving an essential role in coordinating both the immune response and the wound healing process [62, 64].

Macrophages are phenotypically diverse and exhibit a high degree of plasticity which allows them to adapt to the microenvironment in which they reside. However, despite their essential role in tissue homeostasis, dysregulation of macrophage activity may heavily contribute to inflammatory and autoimmune diseases. This is particularly evident in chronic wounds, where inflammatory macrophages play a significant role in the persistence of inflammation and, consequently, affecting wound healing.

In healthy skin, injuries lead to the recruitment of both tissue-resident and monocyte-derived macrophages to the site of injury, where they help orchestrate the three-phase wound healing response. Immediately after an injury occurs, the inflammatory phase begins. Macrophages are polarized to an M1 (inflammatory) phenotype as a means of promoting an anti-pathogenic microenvironment. Following pathogen clearance, macrophages transition towards a spectrum of M2 (inflammation-resolving) phenotype as the wound becomes ready to enter the proliferative phase and, eventually, the tissue-remodeling phase. Because non-healing wounds tend to be caused by the persistence of the inflammatory phase, influencing the behavior of macrophages has been at the forefront of wound healing research. This can be accomplished by either attenuating the activity of M1 macrophages or enhancing the activity of M2 macrophages.

Therapies are often grouped into two broad categories: pharmacological therapies or ex vivo transplantation of macrophages [65]. Ashcroft et al. [66] indicated improved rates of closure in wounds after the application of Infliximab, neutralizing antibodies of TNF-α. Infliximab was applied topically in 8 patients with 14 distinct chronic wounds. Seven of the 8 patients enrolled had positive clinical outcomes, with 12 of the 14 individual wounds responding to treatment. Five treatment-refractory ulcers healed within 4 to 8 weeks, and 4 ulcers showed a reduction in size of 75% after 8 weeks. Non-healed wounds included 2 ulcers which showed no reduction after 8 weeks and a single ulcer that did not display any response to treatment [17, 66].

Pharmacological therapies that stimulate the activity of M2 macrophages have also been explored. Agonists of the peroxisome proliferator-activated receptor, such as GQ-11 have been suggested to improve reepithelization and collagen deposit by repolarizing inflammatory M1 macrophages towards an M2 state in a diabetic murine model. Silva et al. [18] demonstrated that the application of GQ-11 resulted in a 30% increase in wound closure 10 d post-wounding in diabetic mice relative to their vehicle control and pioglitazone groups. It should be noted, however, that this improvement occurred only during the later stages of wound closure and that this positive closure effect was not observed in non-diabetic mice.

Although several disease models show promising findings for macrophage transplantation, the evidence of benefit in wound healing is unclear. Due to the highly concerted and convoluted M1-to-M2 progression, the successful administration of polarized macrophages depends on the appropriate temporal application during the sequence of wound healing. Gu et al. [67] presented evidence on how the administration of activated M1 macrophages within 1 d of injury accelerated wound healing. Moreover, Jetten et al. [68] found that administration of IL-4/IL-10-stimulated M2 macrophages during the early inflammatory phase was detrimental. Dreymueller et al. [69] corroborated these findings, with M1 transplantation sparking the wound healing process when delivered early on, but M2 transplantation significantly delaying the recovery in a chronic wound murine model. These groups concluded that, at least in the context of cutaneous healing in mice, attempting to modify the wound environment through the external introduction of M2-polarized macrophages was not an effective therapeutic approach. Instead, therapeutic strategies directly targeting pro-inflammatory factors like IL-1β or TNF-α appear more promising [68].

The data presented above suggest that SOC treatments, including debridement, disinfection, and appropriate dressing, are more beneficial than macrophage transplantation [69]. There are no FDA-approved drugs to pharmacologically target or transplant macrophages specifically for wound healing, to date. We believe further research is warranted to better understand the complex inflammatory microenvironment of chronic wounds to learn how macrophage transplantation in chronic wounds could become therapeutically viable.

### Fibroblasts

Fibroblasts are a heterogeneous group of mesenchymal cells responsible for creating and maintaining the ECM. The ECM is essential in establishing the internal milieu, providing structural integrity, serving as a growth factor repertoire, and regulating cell activity [70, 71]. Fibroblasts also play a direct role in tissue homeostasis by responding to signaling cues, secreting growth factors and inflammatory cytokines, and having contractility properties. In spite of all of these functions, fibroblasts usually remain in a quiescent state until homeostasis is disturbed [70, 71].

The central role that fibroblasts play in tissue maintenance necessitates that they also orchestrate the return to homeostasis during injury. As a wound occurs and enters the inflammatory phase, fibroblasts exit quiescence and become myofibroblasts, an inflammatory, secretory, and highly contractile phenotype that helps coordinate wound closure. Myofibroblasts migrate into the wound bed, generating large amounts of ECM and releasing cytokines and growth factors. Myofibroblast contraction further helps in wound compaction. As with inflammatory macrophages, dysregulation and persistence of myofibroblast activity can maintain wounds in a chronic, non-healing state [71]. Their central role in regulating, or dysregulating, the wound healing process consequently makes them an attractive therapeutic candidate.

Like the absence of stem cells, the broad depletion of growth factors in chronic wound beds suggests that supplementation could serve as an effective therapeutic. While several growth factors are known to stimulate fibroblast activity, proliferation, and survival, the three following are the most studied: platelet-derived growth factor (PDGF), the fibroblast growth factor (FGF) family, and TGF-β [72]. As demonstrated by Tassi et al. [73], fibroblast growth factor-binding protein 1 (FGF-BP1) enhances angiogenesis, granulation tissue formation, and both fibroblast and keratinocyte migration in their murine wound healing model. A significantly accelerated wound closure rate was observed in transgenic mice that expressed BP1 while FGF2-null mice showed delayed wound closure, suggesting that BP1 plays an important role in FGF activity. By day 6, BP1-expressing wounds were nearly closed while the equivalent control did not demonstrate epithelial closure. And despite the challenges of studying the effects of TGF-β due to its pleiotropic effects, in vitro and in vivo studies provide overwhelming evidence of its necessity for re-epithelialization, inflammation, angiogenesis, and granulation tissue formation during the wound healing process [74].

Despite the successful pre-clinical outcomes, only a handful of growth factor-based therapies have become FDA-approved and are used clinically for chronic wound treatment. In fact, beclapermin, a recombinant human PDGF approved for diabetic neuropathic ulcers, is the only growth factor that is FDA-approved and in-use to date. In contrast to a placebo gel, the application of becaplermin gel at 100 µg/g demonstrated a notable improvement in wound closure outcomes (*n* = 382 patients). Specifically, it increased the rate of complete wound closure by 43% and reduced the time required to achieve complete wound closure by 32%. Adverse events reported during treatment or within a 3-month follow-up period were comparable in terms of type and frequency across all treatment groups [19]. However, there are therapies that are commercially available outside of the United States. These include Fiblast Spray [recombinant human basic fibroblast growth factor (bFGF) available in Japan], Heberprot and Easyef (recombinant human EGF formulations available largely in East Asia), and Regen-D150 (recombinant human EGF gel available in India) [75]. It is postulated that the short half-life, particularly in the presence of the inflammatory microenvironment within chronic wounds, our inability to temporally regulate growth factors, and the relatively limited physiological scope of single growth factor therapies have hampered their clinical use. Research is underway to develop biomaterials that can avoid these barriers and deliver growth factors to chronic wounds more efficiently.

As with other cellular therapies, the direct transplantation of live fibroblasts allows for the regulated release of growth factors and cytokines based on the cells’ ability to sense and respond to the microenvironment. Fibroblasts are often embedded in a scaffold which include growth factors, keratinocytes, or other cellular components that support their survival and ability to improve wound healing [76]. Importantly, some studies have found that both autologous and allogeneic fibroblast-based sheets, whether they are dry, frozen, or non-frozen, had comparable effects on improving wound healing outcomes [77–79]. This is clinically impactful for a few reasons: allogeneic fibroblast sheets are quality-tested and normalized, present considerable time and cost savings, and offer an overall expedited treatment process compared to autologous sheets. Clinically, 8 fibroblast-based cell therapies are in use for wound management to date [80]. Notable examples include: Dermagraft, Apligraf, and TheraSkin [20–22, 80]. A prospective, multicenter RCT found complete wound healing in 34% (*n* = 64) of Dermagraft-treated patients compared to 31% (*n* = 56) of SOC-treated patients. The study concluded that Dermagraft is comparable but not necessarily superior in outcomes relative to SOC therapies [20]. On the other hand, another international, multicenter RCT found that type 1 and 2 diabetes patients undergoing Apligraf treatment showed a significantly higher wound closure rate (*n* = 33 with 51.5% response rate) in the treatment group relative to SOC (*n* = 39 with 26.3% response rate) after 12 weeks [21]. Finally, a multicenter, retrospective, propensity-matched cohort study that analyzed the efficacy of TheraSkin (*n* = 1997) compared to SOC (*n* = 1997), and found that TheraSkin treatment led to a statistically significant increase in healing rate at 90 d, between 90 – 179 d, and beyond 180 d. These data indicate that combined cellular therapies show great promise for the treatment of chronic, non-healing wounds [22].

### Platelets

Platelets are small, anucleated cell fragments that originate from megakaryocytes in the bone marrow and are essential for the wound healing process. Besides being directly responsible for hemostasis, the first phase of wound healing, via plug formation, they also orchestrate the start of the inflammatory phase by serving as adhesion points for recruited neutrophils and monocytes and by secretion of chemokine (C-X-C motif) ligand 4, IL-1β, and other inflammatory mediators [81–83]. Additionally, perhaps most importantly, platelets serve as an enormous source of growth factors, notably PDGF, VEGF, FGF, and EGF [75, 84], which aid in the progression of normal wound healing. Platelet-based therapies, collectively named platelet concentrates, are subsequently based on the knowledge that wounds are depleted of growth factors, that these growth factors can be sourced from platelets, and that platelets contain a variety of growth factors that clinically outperform the use of any given factor by itself.

Platelet concentrates are broadly divided into 4 distinct groups: platelet-rich plasma (PRP), platelet-rich fibrin (PRF), platelet lysate (PL), and platelet extracellular vesicles (PEVs). PRP is a first-generation plasma concentrate derived autologously from a patient’s blood and is the most widely known platelet concentrate [85]. It consists of a plasma fraction with a higher concentration of platelets than whole blood. Its ease of generation, abundance of growth factors, and low immunogenicity make it a popular therapeutic choice in regenerative medicine, particularly in wound healing [85]. Farghali et al. [86] tested subcutaneous PRP infiltration to assess its effect on full-thickness cutaneous wounds. They found that PRP-treated wounds in dogs had higher rates of re-epithelization, increased contractility, more collagen deposition, and acceleration of granulation tissue maturation while also displaying reduced scarring. Clinically, because it is derived from a patient’s blood, PRP is not considered a drug and is therefore not subject to FDA approval. Rather, the use and application of PRP is at the discretion of a patient’s healthcare provider. Its effectiveness in treating chronic wounds, however, is still debated. Qu et al. [87] performed a meta-analysis of RCT and found evidence that autologous PRP may improve healing in diabetic non-healing wounds but insufficient evidence for venous or pressure ulcers.

PRF is a second-generation plasma concentrate that builds and improves upon the success of PRP [85]. The generation procedure is similar to PRP. However, it avoids blood collection tubes coated with anticoagulants and, instead, allows for the formation of a fibrin mesh in the tube in which platelets and leukocytes become embedded in elevated concentrations. The entrapment of cells is thought to prolong and help regulate the pace of growth factor release [85]. Like PRP, PRF is not subject to FDA approval if sourced from a patient’s blood. Pinto et al. [23] designed a prospective, self-controlled study [*n* = 44 patients, with venous leg ulcers (*n* = 28), diabetic foot ulcers (*n* = 9), pressure ulcers (*n* = 5), or complex wounds (*n* = 2)] and observed that all except 5 of the 44 patients with these treatment-refractory chronic ulcers were able to attain wound closure when treated with PRF. Moreover, the 5 remaining patients in which PRF treatment did not completely heal the wound had ulcers > 10 cm^2^ and quit treatment before wound closure. However, their wound recovery was similar to other large wounds that successfully healed, suggesting full recovery was attainable if therapy had not been discontinued. It should be noted that since the study is self-controlled, the strength is limited. However, the group concluded that PRF is a safe, effective, and cheap therapeutic option for these treatment-refractory patients. A more extensive review by Miron et al. [24] found that out of a total of 31 clinical studies, 18 studies (58%) reported positive wound-healing outcomes linked to PRF treatment, even when compared to control groups. Moreover, 27 (87%) out of the 31 clinical studies, supported the utilization of PRF in the context of soft tissue regeneration and wound healing across various medical procedures. This systematic review underscores the favorable impact of PRF on wound healing following regenerative therapy for managing diverse soft tissue issues, including chronic non-healing wounds, encountered in both medical and dental practices.

Finally, PL and PEV are both relatively new and understudied platelet concentrates, relative to PRP and PRF, that have the potential to reach clinical application. PL is generated by freezing/thawing platelets, allowing them to lyse and release their intracellular contents. da Fonseca et al. [88] extensively reviewed the use of PL in different diseases, recommending its use in clinical practice. Additionally, Barsotti et al. [89] broadly characterized the effect of platelet concentrates across several cell types in the wound bed by adding variable PL concentrations. They found that fibroblast migration, keratinocyte epithelization, human umbilical vein endothelial cell viability, proliferation, angiogenic capacity, and monocyte chemotaxis were elevated when exposed to PL. However, it should be noted that Bonferoni et al. [90] compared the effectiveness of PRF to PL, finding that PRF yields better wound healing outcomes. The skew towards PRP/PRF in the literature suggests that these therapies may be more clinically applicable than PL.

PEVs are collected by stimulating platelets and collecting the extracellular vesicles they secrete. Interestingly, PEVs are quite heterogeneous and are amenable to the stimuli applied to the platelets [91]. Guo et al. [92] indicated that exosomes derived from PRP were able to induce migration and proliferation of endothelial cells and fibroblasts to improve wound healing both in vitro and in vivo in a full-thickness wound model in diabetic rats (*n* = 36). Studies regarding their clinical use, however, are still early-stage and sparse. In fact, the first and only human clinical trial that investigated the safety of allogeneic PEVs in non-healing wounds was recently performed by Johnson et al. [25]. Because the group focused on safety, no difference in wound healing capacity was found across experimental and control groups. Rather, they suggest future studies investigate clinical concentrations that are effective for wound regeneration.

## Immunotherapies for wound healing

### Antibodies

**Fig. 3:**
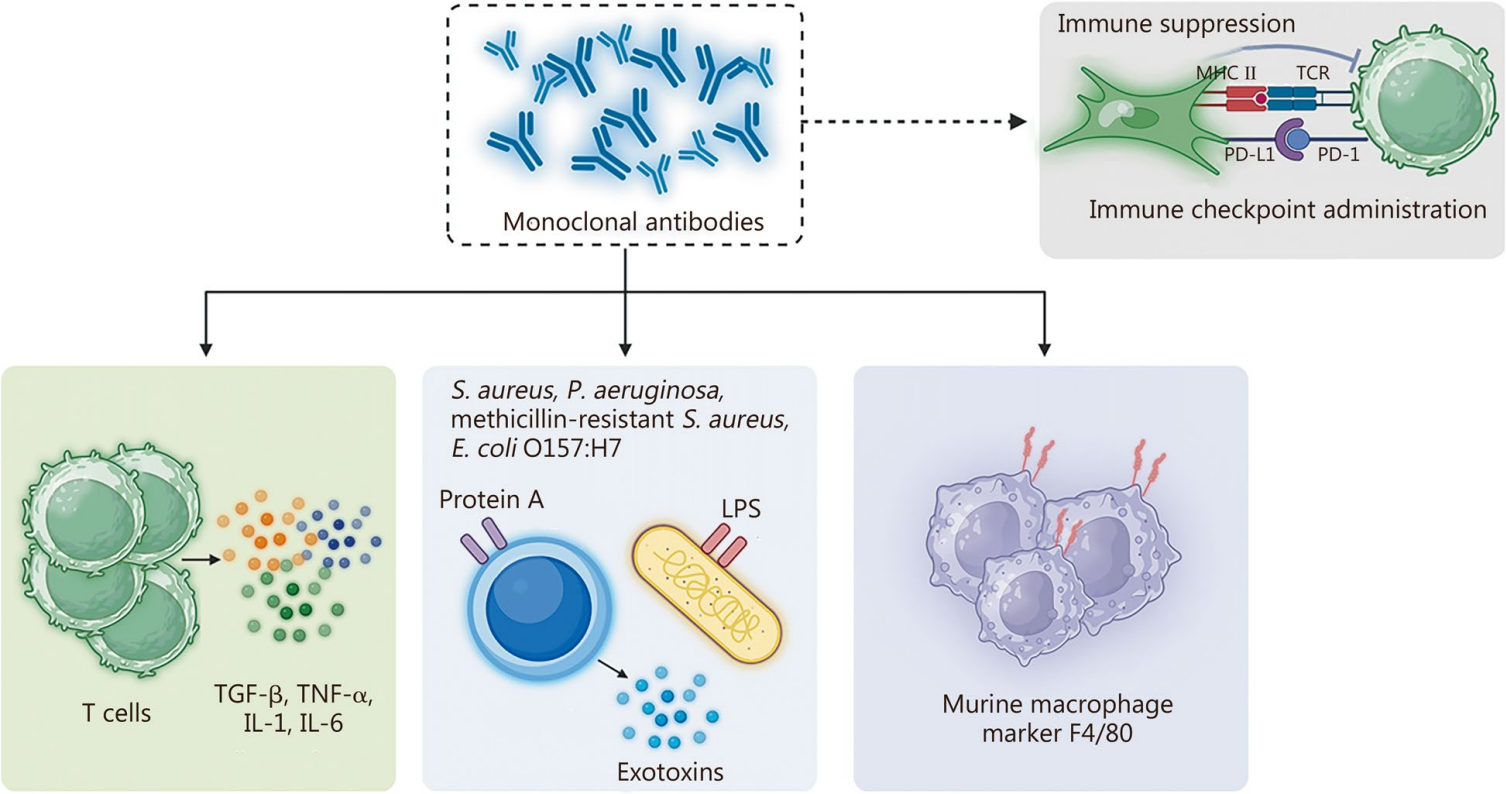
Antibodies represent a versatile platform to target different cells and molecules involved in chronic wound healing. Created with Biorender.com. *S. aureus Staphylococcus aureus*, *P. aeruginosa Pseudomonas aeruginosa*, *E. coli Escherichia coli*, LPS lipopolysaccharide, TGF-β transforming growth factor-β, TNF-α tumor necrosis factor-α, IL interleukin, MHC II major histocompatibility complex class II, TCR T-cell receptor, PD-1 programmed cell death 1, PD-L1 programmed cell death-ligand 1

Monoclonal antibodies have quickly become a leading drug class, with indications across cancer, autoimmune disease, transplant rejection, and infectious diseases (Fig. 3) [93]. According to The Antibody Society, however, there are currently no therapeutic antibodies approved or under review by the FDA for chronic wounds [94].

Acute and chronic wounds contain diverse microbiological populations which may limit wound recovery [95, 96]. We briefly comment below on a prominent use of antibodies: the targeting of bacteria within wound beds. Antibodies against a host of bacterial and fungal elements [97, 98], and exhibit anti-biofilm activity in vitro and in vivo have been reviewed by Watson et al. [99], although the authors deny the present availability of any licensed anti-biofilm antibodies. They explain that anti-biofilm antibody therapies are enhanced by both targeting multiple biofilm components and pairing their use with antibacterial drugs. In combining these two ideas, antibodies conjugated to nanomaterials or antibiotics are becoming extensively studied as a potential therapy for biofilm disruption and elimination [100]. We explore the below antibodies that target pathogenic toxins as well as those conjugated to nanomaterials. Antibodies conjugated to drugs are explored in further detail in the Biofilm section [95, 96].

Antibodies against *Staphylococcus aureus* (*S. aureus*) virulence factors have been shown to have wound healing applications [101–105]. By exploiting the heat generated by the interaction between alternating magnetic field (AMF) and magnetic nanoparticles conjugated with antibody against *S. aureus* protein A, Kim et al. [104] reported an in vitro inactivation of 80% in colony forming units in AMF treatment compared to 50% with an alternative treatment control. The reduction of bacterial burden consequently enhanced wound healing rates and outcomes in AMF-treated mice compared to controls. In a similar vein, Chen et al. [105] used magneto-ovoid strain (MO-1) magnetotactic bacteria and coated them with anti-MO-1 polyclonal antibodies. They successfully targeted the magnetotactic bacteria to *S. aureus* through interactions between *S. aureus* protein A and the antibodies’ Fc fragments. AMF exposure produced magnetic hyperthermia conditions in vitro leading to a 50% increase in the killing efficiency of *S. aureus* in suspension compared to approximately 30% efficiency for AMF-treated, uncoated MO-1 cells [105]. Moreover, significant reductions in wound lengths at 7 d post-wounding were observed in mice receiving treatment of antibody-coated MO-1 with AMF compared to groups who received no treatment, AMF-only, and antibody-coated MO-1 without AMF [105]. In a diabetic murine model of polymicrobial wounds published by Tkaczyk et al. [103], triple monoclonal antibody treatment against multiple *S. aureus* virulence factors (alpha toxin, four secreted bicomponent leukotoxins, and fibrinogen binding cell-surface adhesin clumping factor A) resulted in full skin re-epithelization within 21 d compared to control-IgG, which did not see any wound closure. The polymicrobial wounds also contained *Pseudomonas aeruginosa* (*P. aeruginosa*) and *Streptococcus pyogenes,* whose numbers were significantly decreased with the triple antibody treatment compared to control-IgG [103]. The authors argued that, due to an over-time decline in NETosis activity and pro-inflammatory mediators in diabetic mice polymicrobial skin lesions following treatment, other pathogens were more easily targeted [103]. Antibodies against other bacterial pathogens have been investigated as well. Barnea et al. [26] investigated the healing of *P. aeruginosa-*infected burn wounds in mice using polyclonal antibodies against the N-terminal of *P*. *aeruginosa* type b flagellin. Results of this study showed that infected mice were systemically treated with different regimens of anti-*P. aeruginosa* antibodies had a reduced mortality rate of 0 – 17%, which was comparable to the mortality rate in the imipenem-treated group, and significantly lower than the control mortality rate of 58 – 83%. Moreover, the IgG-treatment group displayed a significantly faster mean wound healing time (15 d vs. 23 d for non-IgG control) with improved histopathological regeneration and no apparent necrosis, ulceration, or abscess formation as was observed in control groups. It should be noted that there were no differences in mortality and wound healing time between anti-*P. aeruginosa* antibody treatment and imipenem antibiotic treatment [26]. Animal studies of wound healing illustrate the promise of bacteria-directed antibodies and their application alongside anti-microbial treatments.

We note that no ongoing clinical trials or FDA-approved antibody therapies for chronic, non-healing wounds are available to date. Rapid proteolysis and clearance of antibodies in inflammatory microenvironments, high costs of manufacturing, potential side effects, and lack of effective delivery systems are all significant challenges that these therapies must overcome if they are to be applied in the clinical setting. However, it is important to highlight those promising innovations in the vehicle space, ranging from nanomaterials to physical and chemical penetration enhancers, that may improve delivery, bioavailability, and persistence in the wound [106–108]. Ultimately, we believe scientific evidence points to the therapeutic potential of antibody-based methods for wound healing.

### Checkpoint inhibitors

Since 2011, there have been 11 immune checkpoint inhibitors approved by the FDA (as of June 2023), all of which are monoclonal antibodies indicated for the treatment of malignancies [94, 109]. The immune checkpoints targeted by these approved checkpoint inhibitors include cytotoxic T-lymphocyte antigen-4, programmed cell death 1 (PD-1), programmed cell death-ligand 1 (PD-L1), and lymphocyte activation gene 3 [94, 109]. There have not yet been any checkpoint inhibitors approved specifically for wound healing indications.

In a study by Afroj et al*.* [110], mouse and human fibrocytes were shown to express PD-L1, and subsequent antibody blockade of the PD-1/PD-L1 interaction enhanced the antigen-presenting and CD8^+^ T-cell-stimulatory activities of these cells. Moreover, Wang et al. [111] have delved into the function of PD-L1 in regulating wound inflammation. They found that PD-L1 expression was detected in mouse fibroblast-like cells within wound granulation tissue. PD-L1 knockout mice used in this study were shown to have delayed healing of excisional wounds, both by gross examination and the relative area of the wound bed that remained over the span of 10 d. Although the most notable disparity in the healed area between wild-type and intervention occurred during days 3, 5 and 7, wild-type mice had healed on average 85% of the excision compared to 70% in PD-L1 knockout mice by the end of the treatment period. Moreover, PD-L1 knockout mice displayed higher expression levels of inflammatory cytokines TNF-α (up to 10% more relative to wild-type) and IL-6 (up to 30% more relative to wild-type). The authors propose that PD-L1 expression of fibroblast-like cells in granulation tissue may positively regulate wound healing via PD-L1-mediated formation of an immunosuppressive microenvironment, initiation of inflammation resolution, and regulation of M1 to M2 macrophage transition [111]. Additionally, TGF-β has been found to play roles in inducing PD-L1 expression of fibroblast and fibroblast-like cells [111, 112]. These findings, when considered together, indicate that inhibition of PD-L1 expressed by fibroblasts may impair wound healing function or reverse the activity of pathological fibroblast phenotypes.

Macrophages and platelets are also involved in wound healing and have been reported to express immune checkpoints in the context of malignancy [113–115]. Immune checkpoint CD83 has been identified as a key mediator of macrophage transition from M1 to M2 phenotypes [116]. Peckert-Maier et al. [116] generated mice with conditional knockout of CD83 expression of macrophages and inflicted full thickness wounds. Compared to wild-type mice, mice with CD83-deficient macrophages displayed an accelerated initial inflammatory wound healing phase, which resulted in an approximately 25% increase in wound closure by day 3. Interestingly, by day 6, both wild-type and CD3 knockout mice had achieved wound closure. Despite this result, histological and expression analysis of CD83 knockout mice revealed an aberrant wound healing process with expanded epidermis, no hair follicle migration, and lacking dermis compared to wild-type mice. Moreover, inflammatory markers such as TGF-β (twofold increase), alpha-actin 2 (38% increase), and type I collagen alpha 1 (40% increase) were elevated in CD83 knockout mice, suggesting that these wounds recovered at least in part via fibrosis [116].

Interestingly, studies have also found that systemic and topical administration, rather than depletion or inhibition, of checkpoint inhibitors have also shown to be beneficial in wound healing. Royzman et al. [117] reported that CD83 application accelerated healing in mouse full thickness excisional wounds compared to control treatment. This group also found that treatment of wounds with macrophages pre-incubated with soluble CD83 improved wound closure compared to phosphate-buffered saline and mock-incubated macrophage treatments, indicating a role for soluble CD83 in modulating macrophage wound healing activity [117]. With respect to PD-L1, Su et al. [118] studied the role of exosomal PD-L1 in vitro and in vivo in mice. In vitro experiments of exosomal PD-L1 administration demonstrated T-cell immunosuppression, which led to enhanced skin cell migration. Topical application of a hydrogel embedded with exosomal PD-L1 on full thickness excisional wounds in mice resulted in complete gross re-epithelization by day 10 compared to control, which still maintained a large scab. Moreover, histological and quantitative polymerase chain reaction analysis revealed markedly reduced levels of inflammatory markers such as TNF-a, IL-6, and granzyme B, as well as diminished immune infiltration, in PD-L1 treatment groups relative to control [118]. Following these findings, Kuai et al. [27] studied external PD-L1 application in a murine model of diabetic ulcers. The authors reported that diabetic ulcers underwent PD-L1 downregulation relative to normal wounds and proposed that endogenous PD-L1 deficiency may be a potential contributor to impaired healing in this context. Administration of topical PD-L1 on ulcers improved healing and re-epithelization rates, even out-performing recombinant bovine basic FGF treatment [27].

The benefits of checkpoint immunotherapy, however, are often accompanied by unacceptable immune-related adverse effects (irAEs). irAEs are common and can manifest in numerous ways, including gastrointestinal, rheumatological, hepatic, neurological, endocrine, adrenal, and cutaneous, amongst many others [119]. Unfortunately, cutaneous irAEs, in particular, are the most common irAEs, which may have implications for wound healing [120]. For example, immune checkpoint inhibitor therapy has been reported to be potentially associated with wound complications in head and neck cancer patients [121, 122]. Notably, the pre-clinical studies listed above did not mention adverse events related to their treatment. We ultimately believe that the pre-clinical evidence warrants further research to evaluate its applicability in human wounds. This should be accompanied by innovation in topical and local delivery systems to avoid systemic irAEs.

### Small molecule inhibitors (SMIs)

SMIs are defined as organic compounds with molecular weights < 500 Da that affect a given biological process. Their small size and oral administration allow them to have low treatment attrition, attain high bio-availabilities, and have the ability to penetrate cell membranes, targeting specific macromolecules to alter their function [123].

Despite the wide-spread use of SMIs in cancer, research regarding their applicability in the treatment of non-healing wounds is sparse. Further, there are no FDA-approved SMIs in use for chronic wounds to date. The few emerging SMI therapies in the literature that have focused on wound healing target the two prominent pathways of the wound healing response: the Wnt and hypoxia inducible factor-1α (HIF-1α) pathways [124].

#### Wnt pathway

Saraswati et al. [28] have assessed the potential of pyrvinium, a Wnt inhibitor, as a wound repair therapeutic. In their study, a polyvinyl alcohol (PVA) sponge implanted subcutaneously in mice was used as a granulation tissue deposition model. They found that pyrvinium-treated PVA sponges had a general improvement in cellular proliferation (150% of Ki-67^+^ expression increase over control), vascularity (120% increase in PECAM-1^+^ expression over control), and overall granulation tissue architecture. Moreover, in a follow-up study, they demonstrated pyrvinium’s potential to enhance MSC proliferation, engraftment, and stemness in an in vivo murine model [29]. These results combined suggest that pyrvinium may be able to serve as an ancillary wound healing therapy.

#### HIF-1α pathway

Groups that target HIF-1α with SMIs have found some evidence of improved tissue repair. Considering HIF-1α is degraded in the presence of oxygen and iron, Thangarajah et al. [30] found that the application of deferoxamine (DFO), an FDA-approved iron chelator, to a humanized, diabetic, murine wound improves perfusion and wound healing outcomes by inhibiting HIF-1α degradation and, consequently, maintaining elevated levels of VEGF. In fact, by day 7, mice treated with DFO had a 13-fold and 2.7-fold increase in CD31 cell and VEGF expression compared to non-treated mice, respectively. These outcomes were further validated by Li et al. [32], who identified the cyclometalated iridium (III) metal complex 1a as a disruptor of the von Hippel-Lindau-HIF-1α protein interaction, helping accumulate HIF-1α in cellulo. Moreover, they showed that wild-type and three distinct diabetic murine models with excisional wounds treated with the iridium (III) complex SMI both showed a significantly accelerated rate of wound healing. In iridium-treated wild-type mice, wound closure was nearly complete by day 8, compared to 75% wound closure in non-treated mice. And across the three diabetic murine models, iridium treatment increased wound closure by up to 25% after 4 d. These changes were accompanied by increased skin thickness, collagen deposition, and importantly, neovascularization. Finally, Parker et al. [31] provide a narrative review that further explores the successes of DFO in pre-clinical models of wound healing and how those results may promote human trials to evaluate its effectiveness in clinical practice.

## Biofilms

**Fig. 4:**
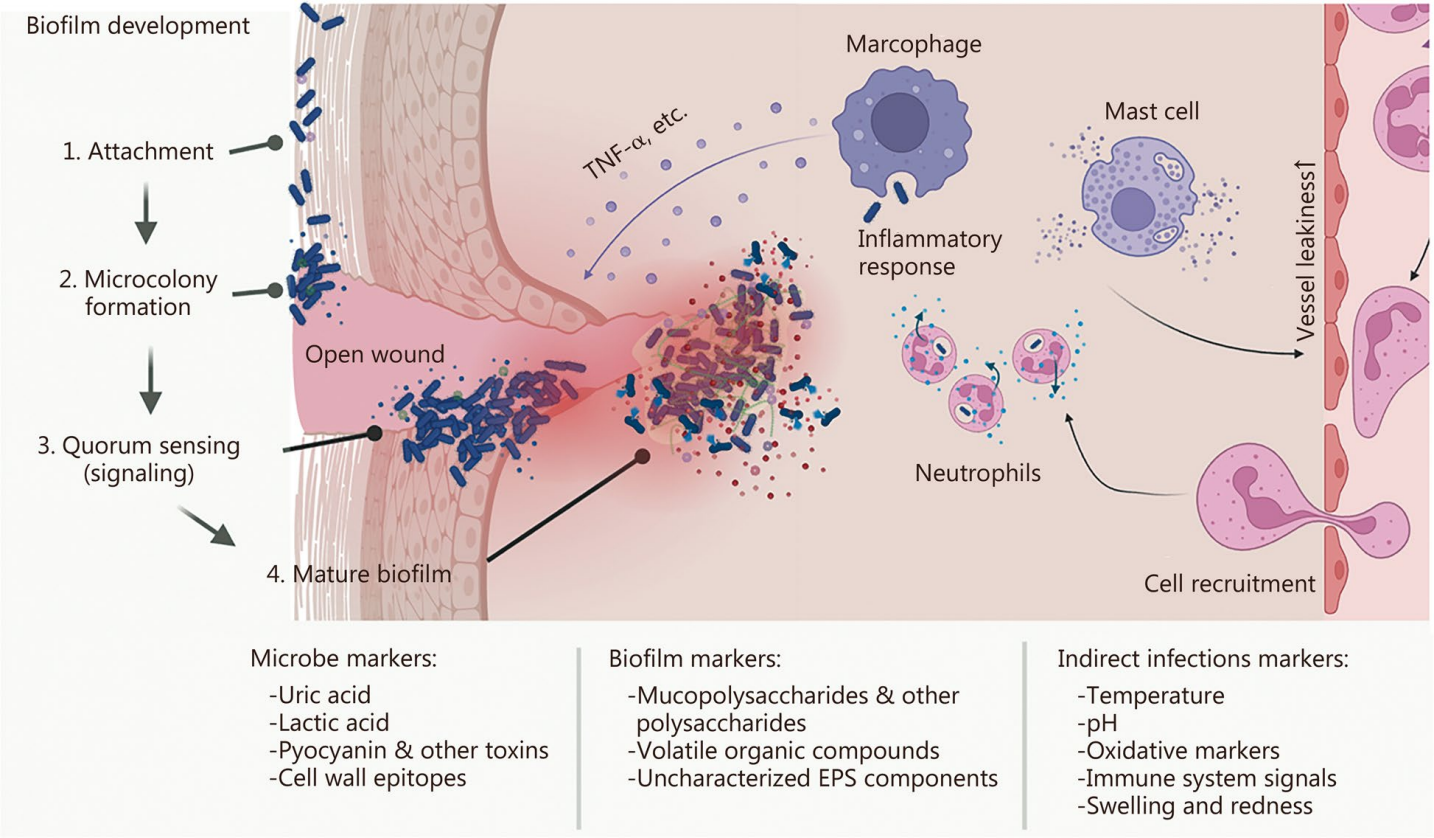
Illustrates biofilm development in an open wound. Attachment of pathogenic germs is followed by microcolony formation that leads to quorum sensing and finally results in biofilm formation. The mature biofilm is resistant to the inflammatory response of the human body due to their extracellular matrix formation. EPS extracellular polymeric substance

Biofilms are complex assemblies of microorganisms embedded in a self-produced ECM that significantly impact wound healing. Primarily composed of bacteria, biofilms create a protective environment for microbial communities, allowing them to resist host immune responses and a variety of antimicrobial treatments [125]. Moreover, while biofilms are present in almost 60% of chronic wounds, they are only present in about 10% of acute wounds [126]. Common biofilm-forming bacteria include *S. aureus*, *P. aeruginosa*, and various subspecies of *Streptococcus* and *Enterococcus*. The presence of biofilms in wounds can lead to prolonged inflammation, delayed healing, and increased risk of systemic infection (Fig. 4) [127]. While there are indeed articles that elucidate the complex interaction between wound healing and biofilm formation, it is surprising that experimental and in vivo wound models that investigate the effects of cellular therapies on wound healing often do not include the consideration of biofilm formation and presence. Therefore, future research is warranted to decipher the bio-pathological mechanisms of biofilm formation in wound beds [128–131]. This lack of consideration may in part explain why promising pre-clinical studies often do not successfully translate into meaningful clinical data.

Tvilum et al. [33] recently developed an anti-*S. aureus* antibody conjugate with mitomycin C, which displayed significant bactericidal properties against *S. aureus* suspensions in vitro. They also assessed its effectiveness against biofilm formations, reporting a 100-fold decrease in colony-forming unit enumeration relative to control and stand-alone vancomycin treatment. However, it was not able to achieve a significant difference against mitomycin C individually, although the group posits that the maximum tolerated dose of the antibody-conjugate was not evaluated. Finally, they reported that the antibody-conjugate formulation of mitomycin C is less toxic in vitro compared to unconjugated mitomycin C. In another study led by Le et al. [34], *S. aureus* biofilms were targeted using an anti-*S. aureus* antibody conjugated to nanoparticle-encapsulated rifampicin. In comparison to phosphate-buffered saline (control)-treated *S. aureus* biofilms, the antibody-nanoparticle conjugate was able to reduce non-biofilm bacterial counts by over 3 orders of magnitude in vitro*.* These findings persisted when applied to a biofilm model, where there was a nearly 140% increase in the relative percentage of live/dead cells compared to control and free-standing rifampicin. Finally, the antibody-nanoparticle conjugate treatment was evaluated in vivo using a prosthesis-associated *S. aureus* biofilm infection murine model and a bioluminescent bacterial strain to quantify bacterial death. Starting at a baseline bioluminescent intensity of 100%, the antibody-nanoparticle conjugate treatment was able to reduce bioluminescence to a mere 5% compared to saline (which saw an increase in bioluminescence to 150%) and free rifampicin (which saw a reduction in bioluminescence to 25%) [34]. Furthermore, Xin et al. [132] have examined the wound healing activity of an ultrasound-activated, antibody-conjugated, perfluoropentane- and meropenem-loaded nanoparticle treatment against *P. aeruginosa* biofilm. The group used a 3D confocal laser scanning microscopy in combination with live/dead staining to qualify the bactericidal effect of treatment, reporting a significant amount of bacterial death in the sonication/nanoparticle group compared to the control, which had maintained high levels of bacteria embedded in biofilm. The treatment was also effective at disrupting biofilm formation in an in vivo murine model. *P. aeruginosa*-infected full-thickness wounds that received the combined sonication/nanoparticle treatment had significantly restricted the bacterial survival rate to 20% at day 3, compared to nearly 100% with control treatment. Moreover, qualitative histological analysis demonstrated complete granulation, hair follicles, and organized connective tissue in treated mice compared to varying levels of inflammation and persistent immune infiltration in other treatment groups including control. Importantly, they concluded their study by assaying for bio-compatibility and safety and found that there was no significant red blood cell lysis or lung, spleen, heart, kidney, or liver inflammation. These results suggest that this treatment may be highly translatable to clinical models.

Limitations, however, do exist with antibody-based therapies against biofilms. Chief amongst them is that biofilms are often composed of several distinct species, current studies seem to achieve similar effectiveness to existing SOCs (although several groups state that the antibody-conjugate titers have yet to be perfected), and the financial burden of antibody synthesis and storage. However, Rembe et al. [9] have tested the application of an in vitro biofilm model consisting of human plasma to cultivate biofilms in a reliable wound model for the laboratory setting. They demonstrated a significantly lower effect of hypochlorous wound irrigation solutions when confronted with biofilm compared to a non-challenged planktonic approach and suggested that this model may be suitable for various applications in biofilm studies. With all this taken into consideration, we believe antibody-based strategies against biofilms are overall supported by literature evidence and continued development will likely yield clinically relevant therapies.

## Conclusions and future perspectives

Traditional wound treatments typically involve the combination of surgical debridement with the application of antiseptics, antibiotics, and dressings to prevent infection and facilitate healing [4]. While effective for managing most wounds, these treatments may have limited efficacy in promoting the healing of chronic and complex wounds. Consequently, we look to innovation in wound therapies to enhance effectiveness in chronic wounds. Cellular therapeutics harness the regenerative potential of living cells, such as stem cells or PRP, to stimulate tissue repair and regeneration. Immunotherapies, on the other hand, modulate the immune response at the wound site using growth factors or cytokines to promote healing and reduce inflammation.

Cellular and immune therapeutics demonstrate great potential in overcoming persistent challenges in chronic wound healing disorders. As for currently available therapies, only a few which FDA-approved, and all of which are cell-based. Results from clinical trials regarding stem cell- and immune cell-based wound dressings have shown promise, but results in this area are often not challenged with the presence or consideration of biofilms. To that extent, pre-clinical results of antibiofilm research are also promising and justify further elucidation. We propose that a multi-pronged approach, in which stem cell delivery and induction provide a matrix for wound remodeling, secretes growth factors, and offers support for other cell types (such as fibroblast proliferation or T-reg recruitment to the wound bed) whilst directly targeting biofilms could be a future avenue of treatment on the way to improve patients’ outcomes and quality of life.

Recent research beyond the scope of biofilms has suggested leveraging inflammation drivers to re-convert the chronic wound to an acute wound microenvironment with elevated pro-inflammatory cytokine and chemokine levels. For instance, Zhu et al. [133] used pro-inflammatory IFN-γ and TNF-α to enrich and amplify the secretome of MSCs. The so-derived supernatant showed promising potential in promoting angiogenesis, constricting collagen deposition, upregulating VEGFC, and accelerating wound closure in a murine cutaneous excision model. Additionally, pro-inflammatory cytokines, including IL-1, IL-17, and TNF-α, have been demonstrated to promote hair follicle neogenesis and epithelialization in chronic wound healing, with IL-1 specifically driving the proliferation and mobilization of stem cells [134]. Overall, these findings highlight the potential positive effects of pro-inflammatory agents on wound healing. Based on these insights, balancing pro-inflammatory vs. anti-inflammatory cytokines and chemokines may represent a promising pathway warranting future research.

In summary, our review sheds light on recent trends and findings on cellular therapeutics and immunotherapies on wound healing. These lines of research may unlock untapped potential and guide further research work. With this in mind, the future of wound care may involve integrating cellular therapeutics and immunotherapies with established treatments to harness synergistic effects and optimize outcomes. Advancements in tissue engineering, regenerative medicine, and immunomodulatory techniques hold promise for developing novel therapies that address the underlying mechanisms of wound healing more effectively. To that effect, we have summarized the mechanism and outcomes of all aforementioned therapies in Table 1. Moreover, although the intended purpose of this paper was to provide a concise but encompassing review of established and developing therapies, we look to other groups to provide more in-depth analyses regarding each therapeutic class. Domaszewska-Szostek et al. [135] provide a comprehensive review of the application and limitations of several different cell-based therapies, including keratinocytes, fibroblasts, BMSCs, and ADSCs, in clinical trials, concluding that although cell-based therapies may in time serve as an alternative to surgical-based treatment, more definitive clinical outcomes are still required. Additionally, Berry-Kilgour et al. [136] extensively detail a review of the use of growth factors and cytokines as immunotherapies for the treatment of chronic wounds. Despite the underwhelming results that cytokine or growth factor clinical trials have had so far, her group notes that as our collective understanding of the physiology underlying chronic wounds improves, tailored growth factor/cytokine therapies, in combination with improved biomaterials and delivery systems, will likely yield improved results.

In conclusion, while traditional wound treatments remain essential for managing acute wounds and preventing infections, cellular therapeutics, and immunotherapies offer exciting opportunities to revolutionize wound care in complex cases. By promoting faster healing, reducing complications, and improving outcomes, these innovative approaches have the potential to significantly impact the field of wound management and improve patient outcomes.

## Data Availability

Not applicable.

## Abbreviations

ADSCs: Adipose-derived stem cells
AMF: Alternating magnetic field
bFGF: Basic fibroblast growth factor
BMSCs: Bone marrow mesenchymal stem cells
CD: Cluster of differentiation
DFO: Deferoxamine
ECM: Extracellular matrix
EGF: Epidermal growth factor
ESC: Embryonic stem cell
FDA: Food and Drug Administration
FGF: Fibroblast growth factor
FGF-BP1: Fibroblast growth factor-binding protein 1
HIF-1α: Hypoxia inducible factor-1α
PSC: Pluripotent stem cells
IFN-γ: Interferon-γ
IL: Interleukin
irAE: Immune-related adverse event
MO-1: Magneto-ovoid strain
MSCs: Mesenchymal stem cells
N/A: Not applicable
PDGF: Platelet-derived growth factor
PD-1: Programmed cell death 1
PD-L1: Programmed cell death-ligand 1
PEVs: Platelet extracellular vesicles
PHD: Prolyl hydroxylase domain
PL: Platelet lysate
PRF: Platelet-rich fibrin
PRP: Platelet-rich plasma
PVA: Polyvinyl alcohol
RCT: Randomized controlled trial
SOC: Standard of care
SMI: Small-molecule inhibitors
TGF-β: Transforming growth factor-β
TNF-α: Tumor necrosis factor-α
T-reg: Regulatory T-cell
VEGF: Vascular endothelial growth factor
